# PRV-delgE/gI/TK immunization preserves blood–brain barrier integrity and limits CNS injury following intracerebral PRV challenge

**DOI:** 10.1128/spectrum.03930-25

**Published:** 2026-05-06

**Authors:** Tong Xu, Baoling Liu, Hanyu Li, Lei Zhao, Yangming Dai, Xin Wu, Siyuan Lai, Jian-Bo Huang, Zhi-Wen Xu, Ling Zhu

**Affiliations:** 1 College of Veterinary Medicine, Sichuan Agricultural University, Chengdu, China; 2 College of Veterinary Medicine, Sichuan Key Laboratory of Animal Epidemic Disease and Human Health, Sichuan Agricultural University, Chengdu, China; Wayne State University, Detroit, Michigan, USA

**Keywords:** pseudorabies virus, intracerebral challenge, blood–brain barrier, neuroprotection

## Abstract

Pseudorabies virus (PRV) causes rapidly progressive and often fatal encephalitis in both natural and non-natural hosts, making the development of effective preventive strategies critically important. Among current vaccine candidates, the triple-gene-deleted PRV strain (PRV-delgE/gI/TK) has been widely studied; however, its protective effects against brain injury, particularly blood–brain barrier (BBB) damage, remain poorly characterized. To address this gap, we established an intracerebral challenge model in C57BL/6J mice to directly evaluate central nervous system (CNS) outcomes following wild-type PRV infection. Wild-type PRV challenge resulted in severe neurological deficits, uniform mortality, extensive neuronal degeneration, pronounced BBB disruption, downregulation of tight junction proteins, strong induction of MMP-2/9, and a robust inflammatory cytokine surge. In contrast, mice immunized with PRV-delgE/gI/TK exhibited only mild and transient symptoms, rapid functional recovery, markedly reduced viral replication, and complete viral clearance by 14 days. Immunization preserved BBB integrity, maintained ZO-1 and occludin expression, prevented MMP activation, and substantially blunted neuroinflammatory responses. Collectively, these results show that PRV-delgE/gI/TK immunization effectively protects against wild-type PRV-induced CNS injury in C57BL/6J mice, as evidenced by reduced viral burden, preserved BBB integrity, and attenuated neuroinflammatory responses, and provide new insight into the neuroprotective effects of attenuated PRV vaccination.

**IMPORTANCE:**

Pseudorabies virus (PRV) can cause severe and often fatal encephalitis, but the central nervous system (CNS)-protective effects of attenuated PRV vaccines remain insufficiently characterized, particularly with respect to blood–brain barrier (BBB) injury. Using a C57BL/6J intracerebral challenge model, this study shows that immunization with the triple-gene-deleted PRV-delgE/gI/TK strain markedly reduces viral replication, preserves BBB integrity, and attenuates neuroinflammatory responses after wild-type PRV challenge. These findings provide *in vivo* evidence that attenuated PRV immunization can protect against PRV-induced CNS injury and offer important insights for improving prevention strategies against neurotropic PRV infection.

## INTRODUCTION

Pseudorabies virus (PRV), a neurotropic alphaherpesvirus, remains a major threat to the global pig industry and has recently attracted increasing attention due to its zoonotic potential (1–3). In non-natural hosts, PRV infection rapidly progresses to severe central nervous system (CNS) lesions characterized by uncontrolled neuroinflammation and fatal encephalitis. With more than 20 human cases reported in the past decade, all associated with occupational exposure to pigs, PRV variants have emerged as pathogens of growing public health concern (4, 5). Understanding the mechanisms of PRV-induced neurological damage and developing effective countermeasures remain important priorities for both veterinary medicine and infectious disease research (2, 6).

The blood–brain barrier (BBB) is a highly specialized barrier system that tightly regulates the movement of substances between the peripheral circulation and the CNS, thereby preserving the stability of the neural microenvironment and supporting normal neuronal activity (7, 8). Anatomically, the BBB is primarily composed of brain microvascular endothelial cells (BMECs), which are supported by a continuous basement membrane, pericytes, and astrocytic endfeet. A key structural feature of the BBB is the presence of tight junctions (TJs) that seal adjacent endothelial cells and limit paracellular diffusion (8–10). These junctional complexes are formed by essential tight junction proteins (TJPs) such as claudins, occludin, and junctional adhesion molecules (JAMs) (10, 11). The cytoplasmic scaffolding protein zonula occludens-1 (ZO-1) links these transmembrane TJPs to the actin cytoskeleton and is critical for maintaining BBB integrity and regulating its dynamic permeability (12).

Increasing evidence indicates that BBB dysfunction is a common pathological hallmark of many viral encephalitides, including infections caused by flaviviruses, herpesviruses, and coronaviruses, highlighting the barrier as a critical interface in viral neuropathogenesis (13). Neurotropic viruses use diverse mechanisms to invade the CNS, and disruption of the BBB is among the most frequent routes of entry. Astrocytes, an essential component of the neurovascular unit, play a central role in maintaining BBB homeostasis by providing structural support and engaging in metabolic and signaling regulation under both normal and pathological conditions (14). Nevertheless, many neurotropic viruses are capable of compromising BBB integrity by interfering with host regulatory pathways, thereby enabling viral penetration into the CNS. For example, several herpesviruses have been reported to induce tight junction disassembly, endothelial activation, and inflammatory signaling that collectively increase BBB permeability and facilitate neuroinvasion (15, 16). A variety of host molecules have been implicated in viral neuroinvasion, including endothelial adhesion molecules such as platelet endothelial cell adhesion molecule (PECAM), CD99, vascular endothelial cadherin (VE-cadherin), and members of the junctional adhesion molecule family (JAM-A, JAM-B, and JAM-C). In addition, chemokines belonging to the CXC, CC, and CX3C families, as well as matrix metalloproteinases (MMPs)—particularly MMP-2 and MMP-9—contribute to BBB breakdown by targeting the basement membrane and tight junction components (17).

Attenuated PRV strains harboring targeted deletions of virulence-associated genes have long been investigated as promising vaccine candidates (18). Among these genes, gE and gI form a functional heterodimer essential for anterograde neuronal transport, while thymidine kinase (TK) is critical for viral replication within neurons (2). Consequently, the triple-gene-deleted strain PRV-delgE/gI/TK has been widely evaluated for its immunogenicity and protective efficacy in both pigs and mice. However, most previous studies have primarily focused on overall protection and immune responses, whereas its protective effects against PRV-induced encephalitis, neuronal injury, and especially BBB damage remain insufficiently characterized. Since BBB disruption is a critical event in PRV-associated CNS pathology, a systematic evaluation of whether PRV-delgE/gI/TK immunization can preserve BBB integrity during wild-type PRV challenge is still lacking.

Based on these considerations, the present study aimed to systematically characterize wild-type PRV-induced neuropathology and BBB disruption in C57BL/6J mice and, using a murine intracerebral challenge model to directly evaluate CNS and BBB outcomes, determine the extent to which prior immunization with PRV-delgE/gI/TK mitigates these changes. We assessed survival, clinical progression, neurological deficits, viral loads, BBB permeability, neuronal integrity, apoptosis, and inflammatory cytokine profiles following PRV challenge. In addition, we analyzed tight junction proteins and matrix metalloproteinases to further examine molecular changes associated with BBB injury and its attenuation after immunization. Collectively, our findings provide a comprehensive evaluation of the CNS- and BBB-protective effects of PRV-delgE/gI/TK immunization in a murine PRV challenge model, deepen our understanding of PRV-induced CNS pathology, and provide useful experimental evidence for improving PRV prevention strategies targeting neuroinvasion and BBB preservation.

## MATERIALS AND METHODS

### Virus, cells, and animals

The PRV-XJ isolate (GenBank accession no. MW893682.1), originally recovered from the brain of a piglet that succumbed to disease on a PRV-vaccinated farm in Sichuan Province, China, was obtained from the virus repository of the College of Veterinary Medicine, Sichuan Agricultural University (Chengdu, China). To ensure experimental consistency, a single stock vial of PRV-XJ was thawed once, dispensed into working aliquots, and stored at −80°C until use.

Specific-pathogen-free (SPF) female C57BL/6J mice (6 weeks old) were sourced from Beijing Huafukang Biotechnology Co., Ltd. (Beijing, China). Animals were housed in individually ventilated cages at 23°C ± 1.5°C with a 12-h light/dark cycle and ad libitum access to food and water. All mice were given a 3-day acclimatization period before experimentation.

### Determination of LD_50_ using an intracranial challenge model

To determine the LD_50_ of the PRV-XJ strain in C57BL/6J mice, viral stocks were serially diluted 10-fold (10^1^–10^7^) in serum-free Dulbecco’s Modified Eagle Medium (DMEM). A total of 48 mice were randomly assigned to seven infection groups (Groups I–VII) and one mock-treated group (Group VIII), with six animals in each group. Mice in Groups I–VII received 20 µL of the respective PRV dilutions via intracranial inoculation, while the mock group was administered an equal volume of DMEM. Clinical signs and mortality were recorded daily for 14 days. The LD_50_ value was calculated according to the Reed–Muench method. In addition, the viral titer of the PRV inoculum used for intracerebral injection was determined as 50% tissue culture infectious dose (TCID_50_) using the Reed-Muench method as previously described (19).

The intracerebral injection procedure was performed as previously described (20, 21). Briefly, mice received an intracerebroventricular injection of 20 μL viral suspension at a controlled rate of 0.2 μL/min. Mice were deeply anesthetized and secured in a stereotaxic apparatus, with the skull maintained in a flat position parallel to the base of the stereotaxic frame. After shaving and disinfection, a midline scalp incision was made to expose the skull, and the injection site was located using the bregma as the reference point. The needle tip was first centered over bregma, and the target site was then determined using stereotaxic coordinates in the anteroposterior (AP), mediolateral (ML), and dorsoventral (DV) planes relative to bregma (AP, 27.2 mm; ML, 57.5 mm; and DV, 16.3 mm from the skull surface), which had been prevalidated in pilot experiments. A small burr hole was drilled at the predetermined coordinates, and the injection needle was slowly lowered to the target depth to minimize tissue trauma. Following completion of infusion, the needle was left in place for approximately 5 min to minimize reflux and facilitate diffusion and then withdrawn gradually. The scalp was subsequently closed, and mice were allowed to recover on a warming pad with postoperative monitoring until fully ambulatory.

### Construction of PRV-infected mouse model

**Fig 1:**
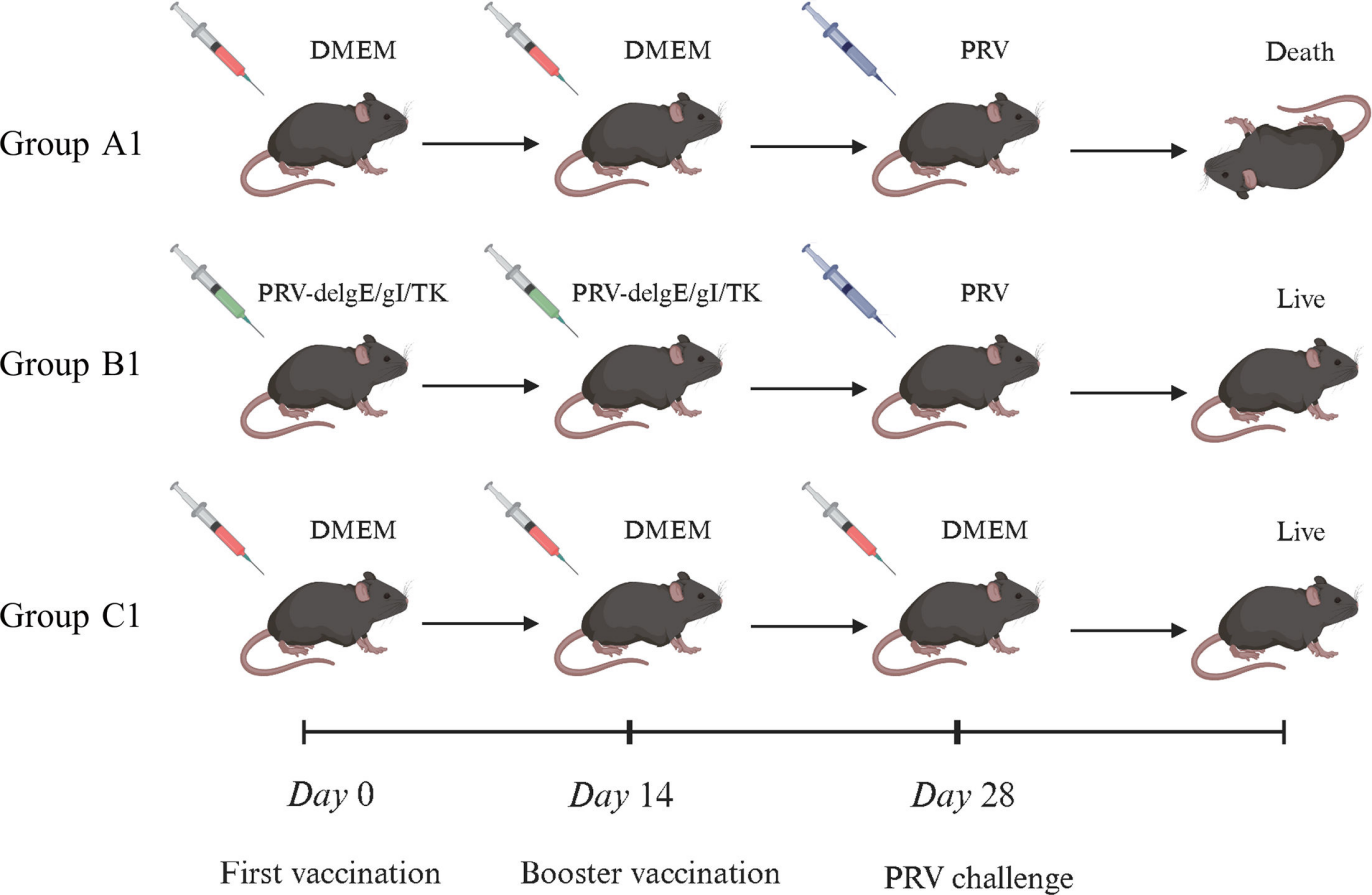
Establishment of the PRV infection model in C57BL/6J mice. Schematic illustration of the experimental design and group allocation: Group A1, PRV-challenged mice; Group B1, PRV-delgE/gI/TK–immunized and subsequently challenged mice; Group C1, mock-treated controls.

Twenty-four 6-week-old female C57BL/6J mice were used to establish the immunization–challenge model. Mice were randomly assigned to three groups: the challenge group (Group A1), the immunization–challenge group (Group B1), and the mock group (Group C1). Each group consisted of eight mice, with four animals allocated to Evans blue extravasation analysis and the remaining four to other assessments. Mice in Group B1 received an intramuscular injection of 0.2 mL PRV-XJ-delgE/gI/TK (10^6^ TCID_50_/mL), followed by a booster immunization 2 weeks later. In contrast, animals in Groups A1 and C1 were administered an equivalent volume of DMEM at both time points. Four weeks after the initial immunization, Groups A1 and B1 were challenged intracranially with 20 µL of PRV-XJ at a dose equivalent to 10 × LD_50_, whereas Group C1 received DMEM via the same route. The experimental design and immunization/challenge timeline are shown in Fig. 1. Mice were monitored daily for changes in clinical condition and survival. Once the final surviving mouse in the challenge-only group reached the moribund stage, all remaining animals across groups were euthanized simultaneously to standardize tissue collection time points.

To investigate the temporal dynamics of PRV replication in the brain following viral challenge, a separate experiment was conducted using 32 6-week-old female C57BL/6J mice. After a 3-day acclimation period, the animals were randomly allocated into two groups: an immunization–challenge group (Group A2) and a mock control group (Group B2) (Fig. S1A). Mice in Group A2 received an intramuscular injection of 0.2 mL PRV-XJ-delgE/gI/TK (10^6^ TCID_50_/mL), whereas mice in Group B2 were administered an equivalent volume of DMEM. At four predetermined time points after challenge—3, 7, 14, and 28 days post-challenge (dpc)—four mice from each group were randomly selected and euthanized for sample collection and quantification of PRV viral loads.

All animals were humanely euthanized by intraperitoneal administration of sodium pentobarbital at a dose of 150 mg/kg. All viral loads in the brain were quantified using a real-time PCR assay targeting the PRV gE gene. Tissue DNA was extracted and amplified using the following primers:

Forward: 5′-CTTCCACTCGCAGCTCTTCT-3′

Reverse: 5′-TAGATGCAGGGCTCGTACAC-3′

Viral copy numbers were calculated using a standard curve with the equation *y* = −3.7938*x* + 43.40, an amplification efficiency of 98.7%, and an *R*² value of 0.998. Viral DNA abundance was calculated using a standard curve and expressed as log_10_ copies per gram of tissue. Viral loads were measured in multiple brain regions, including the medulla, cerebellum, pons, hypothalamus, hippocampus, and cerebral cortex. Different regions of the mouse brain were dissected according to previously described methods (22, 23). Based on preliminary results showing the highest viral burden in the medulla, this region was selected for longitudinal assessment of viral kinetics at 3, 7, 14, and 28 dpc.

### Histopathology, neuronal integrity, and apoptosis analysis

Brains were collected immediately after euthanasia and immersion-fixed in 4% paraformaldehyde at 4°C for at least 24 h. Fixed tissues were embedded in paraffin, sectioned, and subjected to hematoxylin–eosin (HE) staining, Nissl staining, and terminal deoxynucleotidyl transferase-mediated dUTP nick-end labeling (TUNEL) assays. All staining procedures were performed by Chengdu Lilai Biological Co., Ltd. using standard protocols.

For each experimental group, four animals were analyzed (*n* = 4). TUNEL staining was quantified in the hypothalamic region. Images were captured using the same microscope, identical acquisition settings, and the same magnification across all groups. One representative field of view with an identical imaging area was collected per animal. Quantification was performed using ImageJ software (NIH, USA). In each field, TUNEL-positive nuclei were counted and normalized to the total number of nuclei in the same field (e.g., DAPI-positive nuclei). The percentage of TUNEL-positive cells was calculated as (number of TUNEL-positive nuclei/total nuclei) × 100%. Each animal was treated as one biological replicate for statistical analyses.

### Assessment of BBB permeability by Evans blue extravasation

Brains were used to evaluate BBB integrity by Evans blue extravasation, a widely accepted marker of albumin leakage, as previously described with slight modifications (24). Mice were first anesthetized by intraperitoneal injection of sodium pentobarbital (45 mg/kg). Once adequate anesthesia was confirmed, a 2% Evans blue solution prepared in sterile saline was administered intravenously at a dose of 4 mL/kg body weight. The dye was allowed to circulate systemically for 1 h. Subsequently, animals were subjected to transcardial perfusion with ice-cold physiological saline via the left ventricle until the outflow from the right atrium cleared completely, ensuring thorough removal of intravascular dye. Following perfusion, brains were rapidly excised, blotted dry, and weighed. Each hemisphere was then homogenized in 50% (wt/vol) trichloroacetic acid (TCA), with the homogenization volume adjusted proportionally to tissue mass. Homogenates were centrifuged at 10,000 × *g* for 20 min at 4°C, and the resulting supernatants were collected. To deproteinize and clarify the samples, each supernatant was diluted with three volumes of ethanol to yield a final 1:3 mixture of 50% TCA and ethanol. Evans blue content within the samples was measured using either fluorometric detection (excitation at 620 nm and emission at 680 nm) or spectrophotometric absorbance readings at 610–620 nm. The extent of BBB leakage was quantified by normalizing dye concentration to the wet weight of the corresponding brain tissue and expressed as micrograms of Evans blue per gram of tissue.

### Modified neurological severity score

Neurological deficits were assessed using the modified neurological severity score (mNSS), an established composite scale commonly applied in rodent models of CNS injury (25). The mNSS encompasses evaluations of motor coordination, balance, gait, sensory responses, and basic reflexes. At predetermined time points after challenge, each mouse underwent a series of behavioral tests, including the beam balance task, walking initiation, assessment of abnormal movements, tactile and proprioceptive sensory testing, and evaluation of reflexes such as the pinna, corneal, and tail-flexion reflexes. Based on the total score, neurological impairment was categorized within the commonly accepted ranges: animals scoring 1–6 were considered to exhibit mild deficits, those scoring 7–12 demonstrated moderate deficits, and those with scores between 13 and 18 were classified as having severe neurological impairment. All examinations were conducted by investigators blinded to group assignments to ensure objective evaluation.

### Immunohistochemistry and image analysis

Immunohistochemical (IHC) staining was carried out following procedures previously established in our laboratory (26). Paraffin-embedded brain sections were deparaffinized, rehydrated, and subjected to antigen retrieval before incubation with a rabbit monoclonal antibody specific for PRV gB protein (produced in-house; 1:500 dilution). A biotin-conjugated, affinity-purified goat anti-rabbit IgG (Proteintech, Wuhan, China) served as the secondary antibody. Color development was achieved using 3,3′-diaminobenzidine (DAB), and nuclei were counterstained with hematoxylin. Quantitative analysis of IHC staining intensity was performed using the IHC Profiler plugin in ImageJ (26). This tool automatically categorizes pixel intensity into four predefined classes: negative, low positive, positive, and high positive. An H-score was subsequently calculated to reflect the overall staining level using the formula:


H-score=1×(% lowpositive)+2×(%positive)+3×(% highpositive)

yielding a theoretical score ranging from 0 to 300.

### Immunofluorescence analysis

Immunofluorescence (IF) staining was performed on paraffin-embedded brain sections after heat-induced epitope retrieval. Sections were permeabilized using 0.1% Triton X-100 in phosphate-buffered saline (PBS), followed by blocking with PBS containing 1% bovine serum albumin (BSA) and 0.3% Triton X-100. Slides were then incubated overnight at 4°C with primary antibodies (ABclonal, China) targeting Zonula Occludens-1 (ZO-1, A0659), Occludin (A2601), MMP-2 (A11144), and MMP-9 (A0289).

After thorough washing, sections were incubated with Alexa Fluor 488-conjugated donkey anti-rabbit IgG (H+L) (Thermo Fisher, A21206; 1:400) as the secondary antibody. Nuclear staining was performed with DAPI (1 µg/mL) for 10 min. Fluorescent images were captured using a fluorescence microscope under identical exposure settings for all groups.

### Western blotting analysis

Western blotting (WB) was performed according to previously published protocols from our laboratory (27). In addition to the primary antibodies used for the IF analysis, β-actin (AC038, ABclonal, China) was included as a loading control.

### Cytokine quantification by ELISA

Brain tissues were harvested and immediately transferred to ice-cold PBS to maintain sample stability. Each sample was homogenized using a pre-chilled glass tissue grinder and centrifuged at 3,000 rpm for 10–15 min at 4°C. The clarified supernatants were collected for cytokine measurement. Cell culture supernatants, when applicable, were processed in the same manner, with centrifugation at 3,000 rpm for 10 min at 4°C prior to analysis. Concentrations of cytokines—including IL-6, TNF-α, CCL3, CXCL10, MCP-1, IL-4, IL-10, TGF-β, NGF, and GDNF—were determined using commercially available ELISA kits (Jianglai Bio, China; Thermo Fisher Scientific, USA) according to the respective manufacturers’ protocols. Absorbance values at 450 nm were recorded using a microplate reader (Bio-Rad, USA), and cytokine concentrations were calculated from standard curves generated for each assay.

### Statistical analysis

Data processing in this study was performed using GraphPad Prism 9.5.1 software, and statistical differences between the two groups were analyzed by a t-test (Student’s two-tailed unpaired t test), and differences between multiple groups were comparatively analyzed using one-way ANOVA. The schematics shown in Fig. 1 and Fig. S1A were created with BioRender.com. A *P*-value < 0.05 was considered statistically significant, and the results were expressed as mean ± SD, *P < 0.05, **P < 0.01, ***P < 0.001, ****P < 0.0001.

## RESULTS

### Determination of the LD_50_ of intracranially inoculated PRV XJ

PRV XJ virus stock was subjected to 10-fold serial dilutions and intracranially inoculated into C57BL/6 mice across seven challenge groups, with one additional mock control group. Mortality was monitored for 14 days post-inoculation, and the results are summarized in Table 1. All mice in groups I–IV succumbed to infection, whereas partial mortality occurred in groups V and VI, and no deaths were observed in group VII (Table 1). Mice exhibited typical pre-fatal clinical signs, including reduced appetite, ear scratching, and irritation.

**TABLE 1:** Determination of LD_50_ in C57BL/6J mice via intracranial inoculation

Dilution	Number	Death	Survival	Accumulated death	Accumulated survival	Fatality rate (%)
10^−1^	6	6	0	31	0	100.00
10^−2^	6	6	0	25	0	100.00
10^−3^	6	6	0	19	0	100.00
10^−4^	6	6	0	13	0	100.00
10^−5^	6	5	1	7	1	87.50
10^−6^	6	2	4	2	5	28.57
10^−7^	6	0	6	0	11	0

Based on group-wise mortality rates, the LD₅₀ of PRV XJ following intracranial inoculation was calculated using the Reed–Muench method, yielding a value of 10⁻^5.64^ per 0.02 mL. Using this LD_50_ value, a PRV intracranial infection model was established with a challenge dose of 10 × LD_50_, which resulted in 100% mortality in challenged mice, confirming the robustness of the selected dose for model construction. Based on Reed-Muench analysis, the TCID_50_ of the PRV-XJ stock used for intracerebral inoculation was determined to be 10^−6.5^/0.02 mL.

### Establishment of a protective model demonstrating that PRV delgE/gI/TK immunization shields mice from PRV-induced brain injury

**Fig 2:**
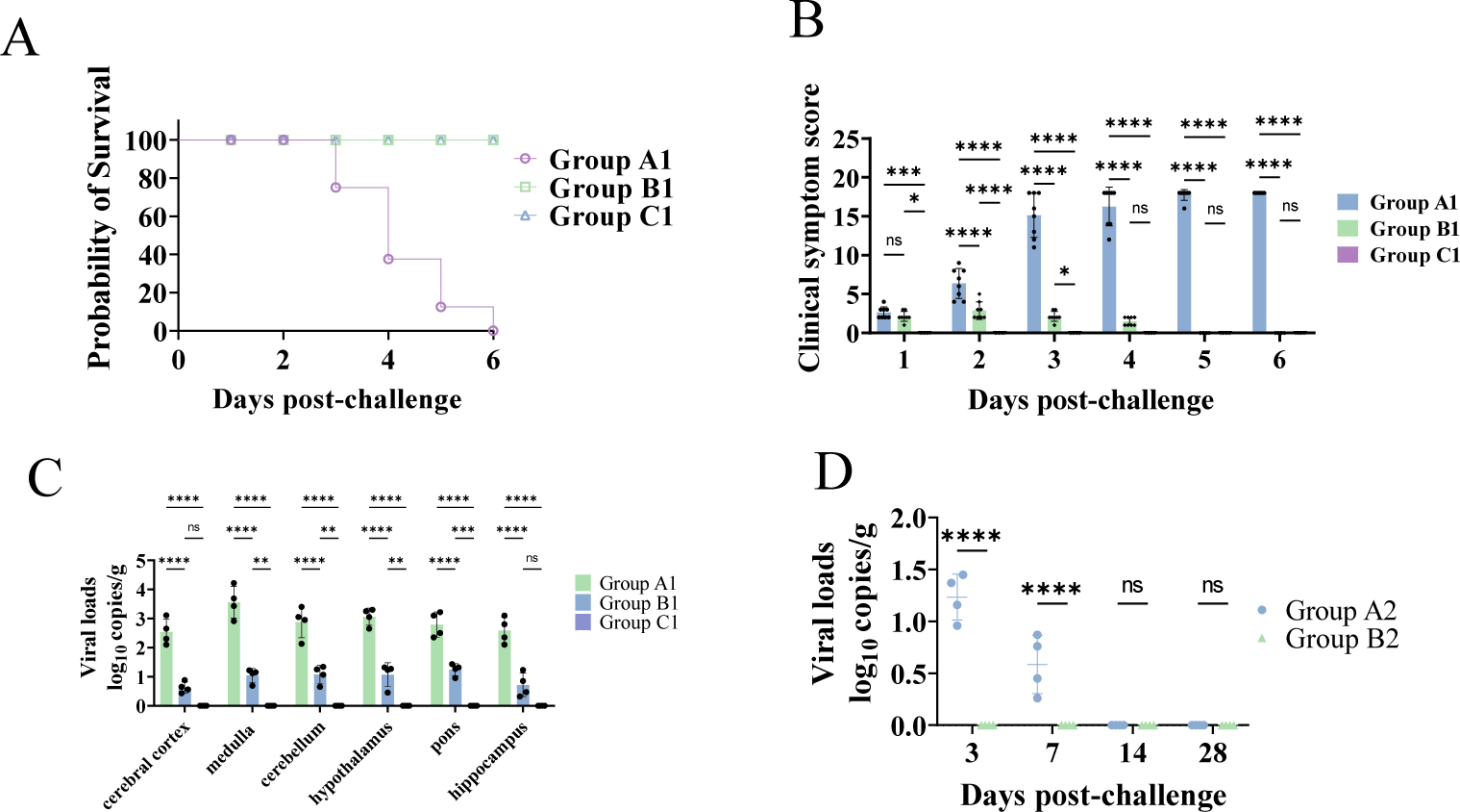
Survival, neurological progression, and brain viral loads in C57BL/6J mice across the three treatment groups. (**A**) Survival curves of the three experimental groups. (**B**) Clinical neurological scores were assessed using the mNSS. (**C**) Viral loads in different brain regions of the challenge and immunization–challenge groups. Samples from the challenge group were collected at the moribund stage, whereas samples from the immunization–challenge group were obtained when the last mouse in the challenge group reached the moribund stage to ensure synchronized comparison. Viral DNA abundance was calculated using a standard curve and expressed as log10 copies per gram of tissue. (**D**) Temporal dynamics of brain viral loads in the immunization–challenge and mock groups at 3, 7, 14, and 28 days post-challenge. Viral DNA abundance was calculated using a standard curve and expressed as log10 copies per gram of tissue. ****P < 0.0001; ***P < 0.001; **P < 0.01; *P < 0.05; ns, not significant.

Two weeks after booster immunization, mice in both the challenge group and the immunization–challenge group were intracranially inoculated with 10 × LD_50_ of wild-type PRV, and clinical signs were monitored continuously. Mice in the challenge group began to exhibit initial symptoms at 48 h post-challenge, including nasal scratching and cage-wall rubbing, which progressively worsened to ruffled fur, hunching, dyspnea, and tremors. All mice in the challenge group succumbed to infection within 144 h (Fig. 2A). In contrast, mice in the immunization–challenge group developed only mild and transient symptoms between 24 and 72 h post-challenge and subsequently recovered completely (Fig. 2A and B). No abnormal signs were observed in the mock controls (Fig. 2A and B).

To further evaluate neurological impairment, mNSSs were assessed at multiple time points. PRV infection induced a time-dependent deterioration in neurological function. At 24 h post-challenge, mNSS did not differ significantly between the challenge and immunization–challenge groups (*P* > 0.05), although both groups showed higher scores than the mock group, with the challenge group displaying a more pronounced increase (*P*<0.01) and the immunization–challenge group showing a moderate elevation (*P* < 0.05) (Fig. 2B). At 48 h, mNSS values in the challenge group were significantly higher than those in both the immunized-challenge and mock groups (*P* < 0.01), and the immunization–challenge group also showed markedly higher scores than the mock controls (*P* < 0.01) (Fig. 2B). By 72 h, deaths occurred in the challenge group, and their mNSS remained significantly higher than those of the other two groups (*P* < 0.01) (Fig. 2B). At 96 h, mNSS values in the immunization–challenge group returned to baseline and were indistinguishable from those of the mock group (*P* > 0.05) (Fig. 2B). Collectively, these findings indicate that neurological decline progressed rapidly in the challenge group, whereas immunized mice experienced only mild transient symptoms with subsequent full recovery.

To compare viral replication within the CNS, viral loads were quantified in multiple brain regions, including the medulla, cerebellum, pons, hypothalamus, hippocampus, and cerebral cortex. Across all examined regions, the challenge group exhibited significantly higher viral burdens than the immunization–challenge group (*P* < 0.01; Fig. 2C), indicating that PRV-delgE/gI/TK immunization markedly suppresses wild-type PRV replication in the brain. Among these regions, the medulla showed the highest viral load and was therefore selected as a representative site for longitudinal analysis. To further characterize viral clearance kinetics, an extended time-course experiment was performed, and brain tissues were collected at 3, 7, 14, and 28 days post-challenge for viral load determination. Viral loads in the immunization–challenge group peaked at 3 dpc, declined progressively thereafter, and were completely cleared by 14 dpc (Fig. 2D). These results indicate that PRV delgE/gI/TK immunization accelerates CNS viral clearance and confers robust protection against PRV-induced brain injury.

### PRV delgE/gI/TK immunization reduces wild-type PRV-induced brain injury

**Fig 3:**
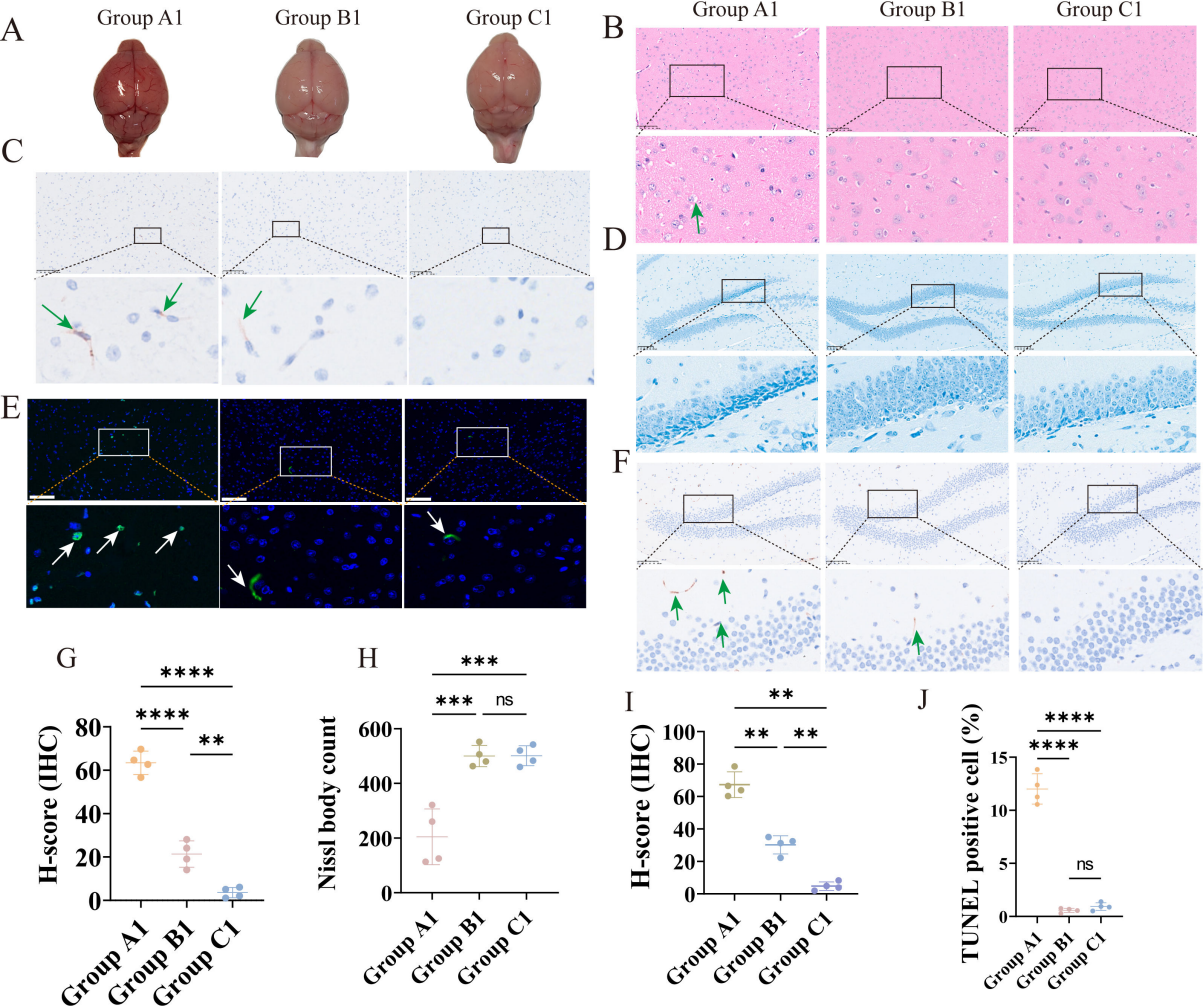
Neuropathological alterations in C57BL/6J mice across the three treatment groups. (**A**) Gross morphology of brains from the challenge, immunization–challenge, and mock groups. (**B**) Representative H&E-stained brain sections (200×; scale bar, 100 μm). Green arrows indicate representative lesion areas. Unless otherwise indicated, all histological images were acquired at 200× magnification with a 100-μm scale bar. (**C**) IHC staining for PRV gB protein in the hypothalamus from the three groups. Green arrows indicate PRV gB-positive regions. (**D**) Nissl staining of hippocampal neurons in the DG region. (**E**) TUNEL staining showing apoptotic cells in the hypothalamus. White arrows indicate areas with positive apoptotic signals. (**F**) IHC detection of PRV gB protein in the hippocampal DG region in the three groups. Green arrows indicate PRV gB-positive signals. (**G**) Quantification of gB immunoreactivity using H-score analysis. (**H**) Quantification of Nissl bodies in the hippocampus. (**I**) Quantification of gB immunoreactivity in the DG region using H-score analysis. (**J**) Percentage of TUNEL-positive cells. ****P < 0.0001; ***P < 0.001; **P < 0.01; *P < 0.05; ns, not significant.

Gross pathological examination revealed marked cerebral congestion and edema, along with prominent meningeal vascular engorgement, in mice from the challenge group, whereas no abnormalities were observed in either the immunization–challenge group or the mock controls (Fig. 3A). To further assess histopathological alterations, H&E staining was performed on brain sections from each group. Mice in the challenge group exhibited pronounced pathological lesions, including parenchymal congestion, hemorrhage, inflammatory cell infiltration, neuronal degeneration and necrosis, and vacuolar changes. In contrast, no apparent abnormalities were detected in the immunization–challenge or mock groups (Fig. 3B).

IHC staining for PRV gB in the hypothalamus revealed strong positive signals in the challenge group, weak positivity in the immunization–challenge group, and no detectable staining in the mock group (Fig. 3C). Quantification using ImageJ H-score analysis confirmed that staining intensity was significantly higher in the challenge group than in the other two groups (Fig. 3G). To determine the kinetics of viral clearance following intracranial challenge in the immunization–challenge group, brain tissues were collected at 3, 7, 14, and 28 dpc for gB IHC. The H-score exhibited a clear time-dependent decline, with significant reductions from 3 to 7 dpc and from 7 to 14 dpc (*P* < 0.01). By 14 dpc, the H-score had decreased to levels indistinguishable from those of the mock group (Fig. S1B and C). These observations were consistent with qPCR viral load measurements, indicating that PRV delgE/gI/TK immunization facilitates time-dependent viral clearance, achieving complete elimination by 14 dpc.

To evaluate neuronal survival following infection, Nissl staining was performed (Fig. 3D and H). Compared with the immunization–challenge and mock groups, mice in the challenge group showed marked neuronal shrinkage, widened intercellular spaces, intensified staining, and a significant reduction in the number of Nissl bodies in the dentate gyrus (DG) region (*P* < 0.01). No significant differences were observed between the immunization–challenge and mock groups (*P* > 0.05). To determine whether the DG region was infected by PRV in different groups, IHC was performed (Fig. 3F). The results showed positive signals in both the challenge and immunization–challenge groups, whereas no signal was detected in the mock control group. Moreover, the positive signal in the immunization–challenge group was significantly lower than that in the challenge group (Fig. 3I). These findings indicate that the DG region is susceptible to PRV infection and that PRV-delgE/gI/TK immunization markedly reduces viral burden in this region.

Neuronal apoptosis in the hypothalamus was then assessed using TUNEL staining (Fig. 3E and J). The number of TUNEL-positive cells did not differ between the immunization–challenge and mock groups (*P* > 0.05), whereas the challenge group exhibited a dramatic increase in apoptotic neurons compared with both groups (*P* < 0.01). Collectively, these findings demonstrate that wild-type PRV induces severe brain pathology, neuronal loss, and apoptosis, while immunization with PRV delgE/gI/TK confers substantial protection against PRV-mediated brain injury.

### Immunization with PRV delgE/gI/TK alleviates wild-type PRV-induced blood–brain barrier disruption

**Fig 4:**
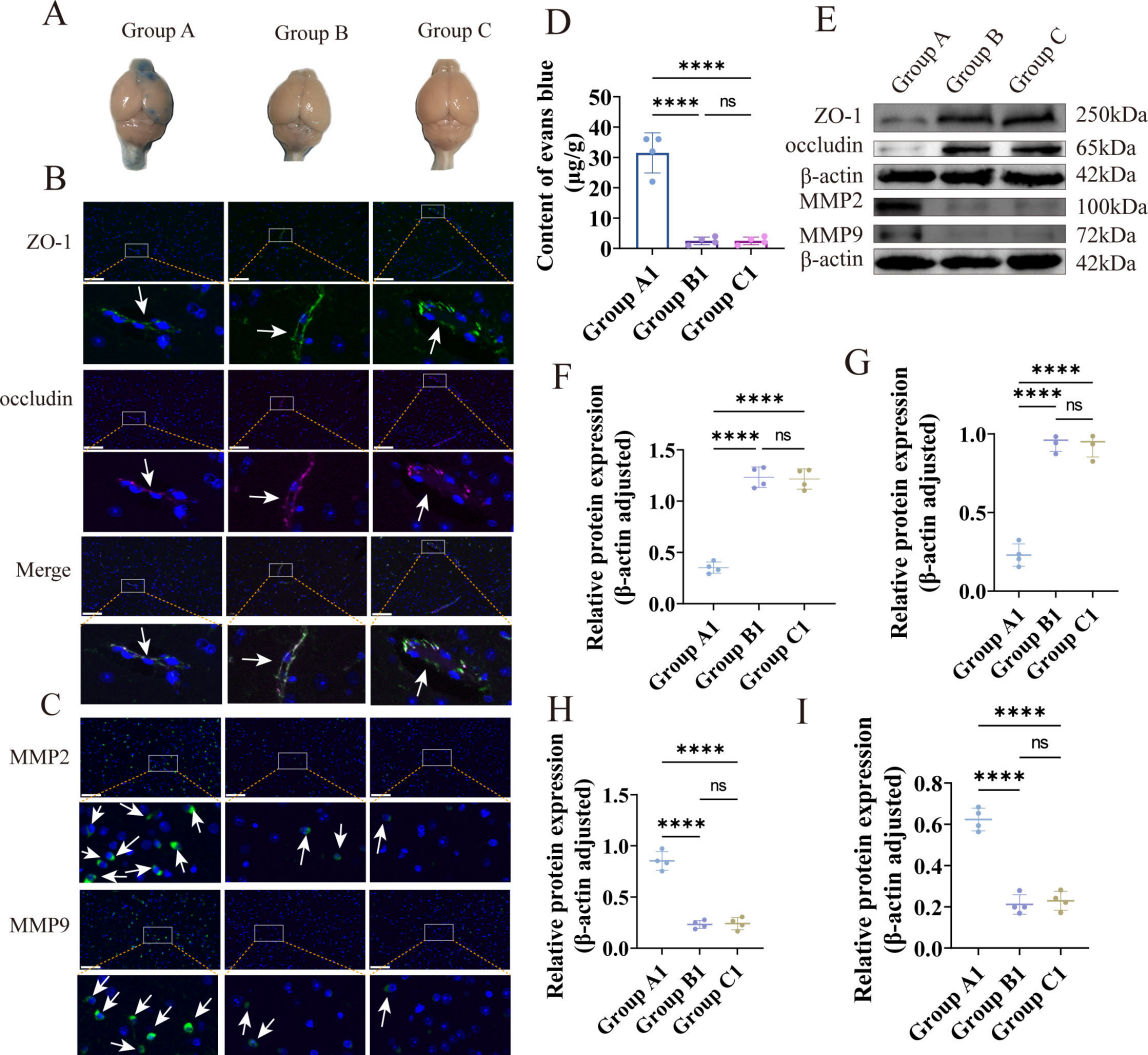
Assessment of BBB disruption in C57BL/6J mice across the three treatment groups. (**A**) Representative gross images of brains collected 45 min after intravenous Evans blue administration and transcardial perfusion showing extensive dye extravasation in the challenge group, whereas minimal or no staining was observed in the immunization–challenge and mock groups. (**B**) IF staining of cortical sections demonstrates the expression of tight junction proteins ZO-1 and occludin across the three treatment groups. White arrows indicate the regions of tight junction proteins in vascular endothelial cells. (**C**) IF staining for MMP-2 and MMP-9, two matrix metalloproteinases associated with BBB degradation, showed marked upregulation in the challenge group. White arrows indicate the regions of positive signal. (**D**) Quantification of Evans blue content in brain tissues indicates significantly increased BBB permeability in the challenge group compared with the other groups. (**E**) WB analysis of ZO-1, occludin, MMP-2, and MMP-9 protein levels in brain tissues collected post-challenge. (**F–I**) Densitometric quantification of WB bands for ZO-1, occludin, MMP-2, and MMP-9, respectively, based on ImageJ analysis. ****P < 0.0001; ***P < 0.001; **P < 0.01; *P < 0.05; ns, not significant.

To evaluate BBB injury among the three treatment groups, BBB permeability was first assessed using the Evans blue extravasation assay. Following intravenous injection of Evans blue for 45 min and subsequent transcardial perfusion, extensive blue discoloration was observed in the brains of mice in the challenge group, whereas no blue staining was detected in either the immunization–challenge group or the mock controls (Fig. 4A). Quantification of Evans blue content revealed a markedly higher dye accumulation in the challenge group compared with both the immunization–challenge and mock groups (*P* < 0.01; Fig. 4D), consistent with the gross observations. These findings indicate severe BBB disruption following wild-type PRV challenge, while immunization with the triple-gene-deleted strain effectively preserved BBB integrity.

Given the critical role of endothelial tight junctions in maintaining BBB function, we next examined the expression of key tight junction proteins ZO-1 and occludin using IF and WB analysis. WB results showed no significant differences in ZO-1 or occludin expression between the immunization–challenge and mock groups (*P* > 0.05). In contrast, both proteins were markedly downregulated in the challenge group (*P* < 0.01; Fig. 4E through G). IF further confirmed these findings, with pronounced loss of tight junction signals in the challenge group but preserved expression in the immunization–challenge and mock groups (Fig. 4B). These results demonstrate that wild-type PRV significantly disrupts BBB structure by reducing tight junction protein levels, whereas immunization with PRV delgE/gI/TK provides robust protection.

MMPs, particularly MMP-2 and MMP-9, are known to degrade tight junction components and contribute to BBB breakdown. To assess their involvement in PRV-mediated BBB injury, we examined MMP-2 and MMP-9 expression in brain tissues. WB analysis showed no significant differences between the immunization–challenge and mock groups, whereas both MMP-2 and MMP-9 were markedly upregulated in the challenge group (*P* < 0.01; Fig. 4E, H, and I). Immunofluorescence staining exhibited the same pattern, with elevated MMP signals in the challenge group but no observable changes in the immunization–challenge or mock groups (Fig. 4C). These findings indicate that wild-type PRV induces strong upregulation of MMP-2 and MMP-9, contributing to tight junction degradation and BBB disruption, while immunization with the triple-gene-deleted strain effectively suppresses MMP-driven BBB damage.

### PRV delgE/gI/TK immunization attenuates the PRV-induced neuroinflammatory storm

**Fig 5:**
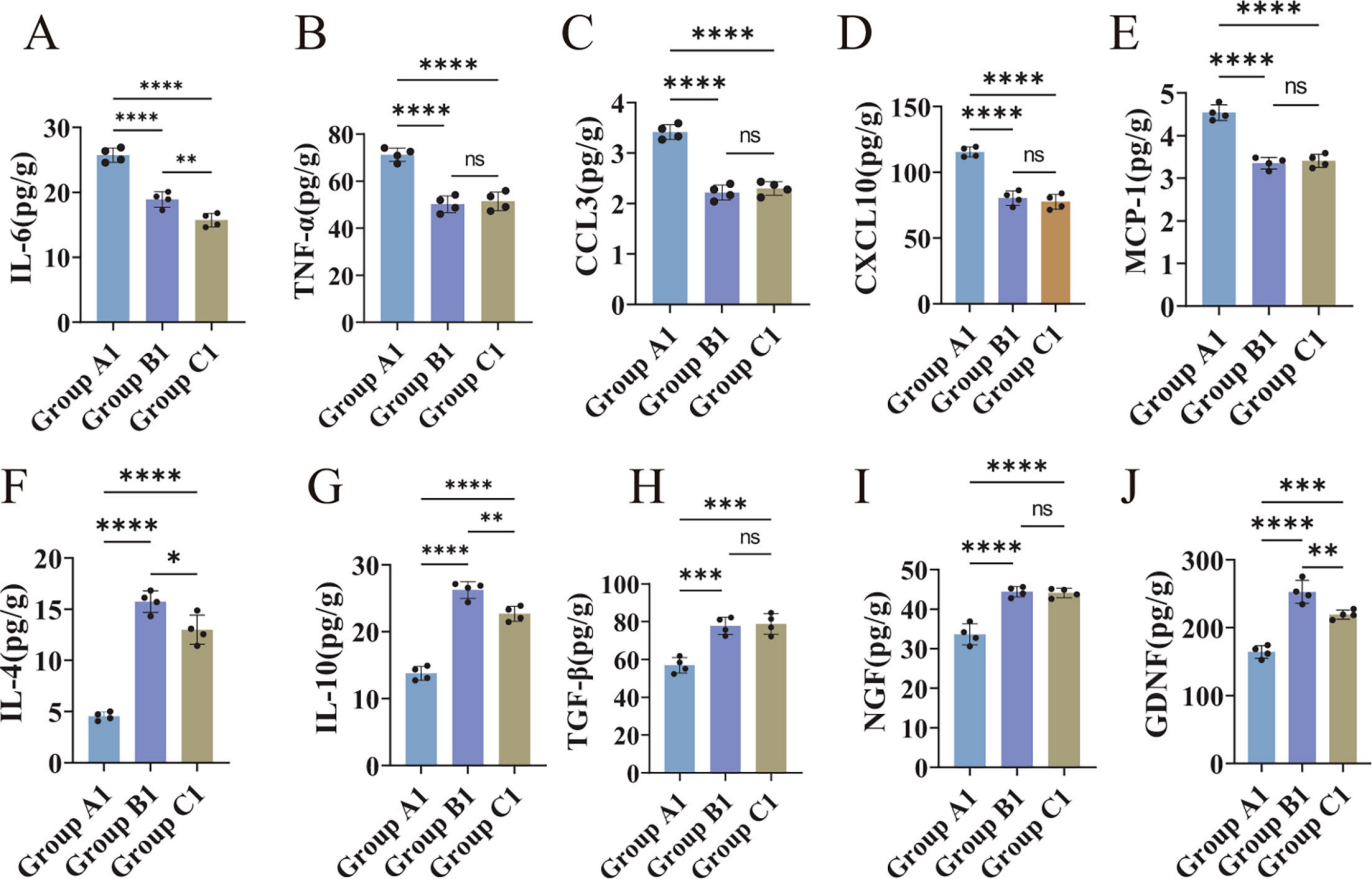
Cytokine and chemokine profiles in C57BL/6J mice across the three treatment groups. (**A–E**) Quantification of IL-6, TNF-α, CCL3, CXCL10, and MCP-1 levels in brain tissue from the challenge, immunization–challenge, and mock groups. (**F–J**) Quantification of IL-4, IL-10, TGF-β, NGF, and GDNF levels in brain tissue from the challenge, immunization–challenge, and mock groups. Data are presented as mean ± SD. Statistical significance: ****P < 0.0001; ***P < 0.001; **P < 0.01; *P < 0.05; ns, not significant.

To determine whether PRV infection triggers excessive neuroinflammation and whether immunization with PRV delgE/gI/TK can attenuate this response, we quantified a panel of pro-inflammatory and anti-inflammatory cytokines, chemokines, and neurotrophic factors in brain homogenates from the three treatment groups using ELISA. As shown in Fig. 5, levels of IL-6, TNF-α, CCL3, CXCL10, and MCP-1 were markedly elevated in the challenge group compared with both the immunization–challenge group and the mock controls (*P* < 0.01; Fig. 5A through E). In the immunization–challenge group, IL-6 remained significantly higher than in the mock group (*P* < 0.01), whereas TNF-α and the three chemokines showed no significant differences between the two groups (*P* > 0.05). Conversely, the anti-inflammatory cytokines IL-4 and IL-10 and the neurotrophic factors TGF-β, NGF, and GDNF were significantly reduced in the challenge group relative to both the immunization–challenge and mock groups (*P* < 0.01; Fig. 5F through J). In contrast, IL-4 and IL-10 levels were significantly (*P* < 0.05) and highly significantly (*P* < 0.01) elevated, respectively, in the immunization–challenge group compared with the mock controls, and GDNF expression was also markedly increased (*P* < 0.01). No significant differences in TGF-β or NGF expression were observed between the immunization–challenge and mock groups.

To further examine cytokine dynamics following challenge, brain tissues from the immunized-challenge and mock groups were collected at 3, 7, 14, and 28 dpc. Compared with mock controls, IL-6 levels in the immunization–challenge group showed a pronounced increase at 3 dpc (*P* < 0.01) before declining to baseline by 14 dpc (*P* > 0.05; Fig. S2A). TNF-α exhibited a similar pattern, with a sharp elevation at 3 dpc (*P* < 0.01) and a return to normal levels by 7 dpc (Fig. S2B). CCL3 and CXCL10 remained comparable between the two groups across all time points (*P* > 0.05; Fig. S2C and D), whereas MCP-1 showed a modest increase at 3 dpc (*P* < 0.05) and returned to baseline by 7 dpc (Fig. S2E). For anti-inflammatory cytokines, IL-4 and IL-10 levels were markedly elevated in the immunized-challenge group at 3 dpc (*P* < 0.01), and remained significantly higher at 7 dpc (*P* < 0.05 and *P* < 0.01, respectively). IL-4 returned to normal levels by 14 dpc, whereas IL-10 normalized by 28 dpc (Fig. S2F and G). Among neurotrophic factors, TGF-β showed no significant difference at any time point (Fig. S2H). NGF and GDNF were both significantly elevated at 3 dpc (*P* < 0.01); NGF declined to baseline by 7 dpc, while GDNF remained elevated at 7 dpc and returned to control levels by 14 dpc (Fig. S2I and J).

Overall, these findings demonstrate that immunization with PRV delgE/gI/TK markedly attenuates the neuroinflammatory storm induced by wild-type PRV, characterized by blunted pro-inflammatory cytokine responses and enhanced or preserved anti-inflammatory and neurotrophic signaling, thereby effectively mitigating PRV-associated neuroinflammation.

## DISCUSSION

PRV, characterized by pronounced neurotropism, rapid replication, and the ability to cause acute and lethal encephalitis in non-natural hosts, continues to pose a substantial threat to animal health and public health (28). Elucidating the molecular mechanisms underlying PRV-induced CNS injury and identifying effective protective strategies remain major priorities in PRV research. Although the triple-gene-deleted PRV strain (PRV-delgE/gI/TK) has been widely investigated for its immunogenicity and protective efficacy, most previous studies have focused primarily on general protection outcomes, whereas its effects on PRV-induced CNS pathology—particularly BBB injury—have been insufficiently characterized. Given the central role of BBB disruption in viral neuroinvasion and neurological damage, a more detailed evaluation of whether PRV-delgE/gI/TK immunization can preserve BBB integrity, limit CNS injury, and attenuate neuroinflammatory responses during wild-type PRV challenge is needed.

Against this background, defining the extent of BBB disruption caused by PRV infection and determining how the triple-gene-deleted strain PRV-delgE/gI/TK protects the CNS against wild-type PRV challenge are of considerable scientific and practical importance. In this study, we employed a rigorous intracerebral challenge model to directly evaluate post-immunization BBB integrity, viral replication dynamics in the brain, neuronal injury, and cytokine responses. Through this approach, we sought to provide a more comprehensive understanding of the CNS- and BBB-protective effects associated with PRV-delgE/gI/TK immunization and to generate new evidence relevant to the protective actions of attenuated PRV vaccine strains.

Neuropathological analyses further revealed that PRV-delgE/gI/TK immunization markedly attenuated neuronal injury. Wild-type PRV caused extensive neuronal degeneration, apoptosis, and cortical and hippocampal pathology, as shown by HE, Nissl, and TUNEL staining. Immunized mice displayed minimal histopathological alterations, and IHC staining confirmed dramatically reduced viral antigen levels in the CNS. Time-course measurements revealed that viral loads in immunized mice quickly declined and became undetectable by 14 days post-challenge, demonstrating efficient viral clearance.

The BBB serves as a highly selective physiological interface that regulates molecular transport between the circulation and the central nervous system, ensuring an optimal environment for neuronal activity and brain metabolism (9, 29). The Evans Blue extravasation assay is widely employed in rodent studies as a reliable indicator of blood–brain barrier disruption and alterations in vascular permeability (24). Our Evans blue assays clearly showed that wild-type PRV induced severe BBB leakage, whereas immunized mice maintained intact barrier function. These findings are consistent with previous reports that neurotropic viral infections commonly target BBB integrity as a key step facilitating CNS invasion (16). Compromise of tight junction structures is a well-recognized feature in models of viral encephalitis and represents a critical step that permits peripheral immune cells and invading viruses to access the CNS (12). Such barrier impairment is commonly associated with substantial reductions in the tight junction proteins ZO-1 and occludin (30–32). In this study, PRV challenge significantly decreased the expression of tight junction proteins ZO-1 and occludin, consistent with structural compromise of the BBB. In contrast, PRV-delgE/gI/TK immunization preserved tight junction integrity.

MMP-2 and MMP-9, the major gelatinases responsible for degrading extracellular matrix components and tight junction structures, are frequently elevated during neuroinflammatory processes and are recognized as important indicators of BBB disruption in viral CNS infections (16, 33). In our study, WB and IF analyses confirmed a marked induction of both MMPs following wild-type PRV challenge, consistent with the severe BBB breakdown observed in this group. Moreover, these findings are consistent with previous reports demonstrating that PRV infection markedly upregulates MMP-9 expression and contributes to BBB disruption (16). Notably, prior immunization with PRV-delgE/gI/TK effectively suppressed the upregulation of MMP-2 and MMP-9, indicating that vaccination helps preserve BBB integrity by preventing excessive enzymatic degradation of barrier structures.

Neuroinflammation also played a critical role in PRV-induced CNS injury. Previous studies have shown that the early neuroinflammatory events of PRV infection are rapidly initiated; for example, PRV has been reported to prime dorsal root ganglia neurons toward an inflammatory state shortly after footpad inoculation (34). Inflammatory responses are typically driven by cytokines such as TNF-α, IL-4, IL-6, and IL-10, which are produced by endothelial cells, glial cells, and neurons and serve as important triggers of CNS inflammation and neuronal damage (35). Moreover, cytokine storms have been associated with the histopathological changes induced by PRV, with reports showing elevated IFN-α, IFN-γ, IL-6, IL-18, and IL-1β expression in the brains of infected mice (36, 37). Consistent with this, wild-type PRV infection triggered a pronounced inflammatory cytokine storm, with sharp increases in IL-6, TNF-α, CCL3, CXCL10, and MCP-1, along with marked suppression of anti-inflammatory cytokines (IL-4, IL-10) and neurotrophic factors (TGF-β, NGF, GDNF). These dysregulated cytokine responses likely amplify neuronal damage and accelerate disease progression. In striking contrast, PRV-delgE/gI/TK immunization blunted the pro-inflammatory cytokine surge while sustaining or enhancing anti-inflammatory and neurotrophic signaling. Importantly, cytokine levels in immunized mice returned to baseline more rapidly than those in the infected group, reflecting effective regulation of the CNS inflammatory environment.

This study primarily focused on the protective effects of PRV-delgE/gI/TK immunization against PRV-induced CNS injury following intracerebral challenge, with particular emphasis on BBB damage. Given that antibody levels in the brain are generally low under physiological conditions, together with the robust protection observed after PRV-delgE/gI/TK immunization in our model, it is tempting to speculate that additional protective pathways may contribute to limiting PRV-induced CNS injury. At present, however, this remains a hypothesis and requires further investigation. Future studies incorporating experimental strategies to minimize or control antibody interference, together with more targeted mechanistic analyses, will be necessary to clarify the relative contributions of different protective components.

Several aspects of the intracerebral challenge model warrant further investigation. First, although the present study demonstrated robust neuroprotection following PRV-delgE/gI/TK immunization, the spatiotemporal dynamics of viral dissemination across different brain regions after intracerebral inoculation were not systematically characterized. A more detailed mapping of viral distribution and regional protective effects would provide deeper insight into the mechanisms of CNS involvement and vaccine-mediated protection. Second, the intracerebral injection volume (20 μL) used in this study was selected based on previously reported protocols but was not independently optimized. Whether this volume represents the optimal dosing condition for reproducible CNS infection while minimizing procedural variability remains to be determined. Future studies exploring dose–volume parameters may help further refine this model. Third, natural routes of PRV exposure and neuroinvasion also warrant further investigation. In particular, intranasal inoculation has been widely used and is highly relevant to natural neuroinvasion. Previous studies, including our own work and those of others, have shown that immunization can effectively protect against PRV-induced brain injury following intranasal challenge (2, 38, 39). In this setting, antibody-mediated immunity, especially mucosal immunity, plays an important protective role (2, 38, 39). In addition, ocular inoculation may represent another biologically relevant route of CNS entry. Although this route has been less extensively studied, it may provide additional insight into alternative pathways of PRV neuroinvasion and therefore deserves further investigation in future studies.

In summary, these findings demonstrate that PRV-delgE/gI/TK immunization provides robust protection against PRV-induced CNS injury by limiting viral replication, preserving blood–brain barrier integrity, and attenuating neuroinflammatory responses. These results deepen our understanding of PRV neuropathogenesis and provide valuable insights for the development of safer and more effective PRV vaccines and therapeutic strategies targeting CNS invasion.

## Data Availability

The data are included in the article and the Supplemental figures. Further inquiries can be directed to the corresponding author.

## SUPPLEMENTAL MATERIAL

Supplemental figures spectrum.03930-25-s0001.docx Fig. S1 and S2.

ASM does not own the copyrights to Supplemental Material that may be linked to, or accessed through, an article. The authors have granted ASM a non-exclusive, world-wide license to publish the Supplemental Material files. Please contact the corresponding author directly for reuse.
