# Supplementary material for: PRV-delgE/gI/TK immunization preserves blood–brain barrier integrity and limits CNS injury following intracerebral PRV challenge
Source: Microbiol Spectr. 2026 May 6;14(6):e03930-25. doi: 10.1128/spectrum.03930-25 (PMC13228039; doi:10.1128/spectrum.03930-25)
Supplement: Supplemental figures — Fig. S1 and S2. [file spectrum.03930-25-s0001.docx]

**Supplementary Figures:**


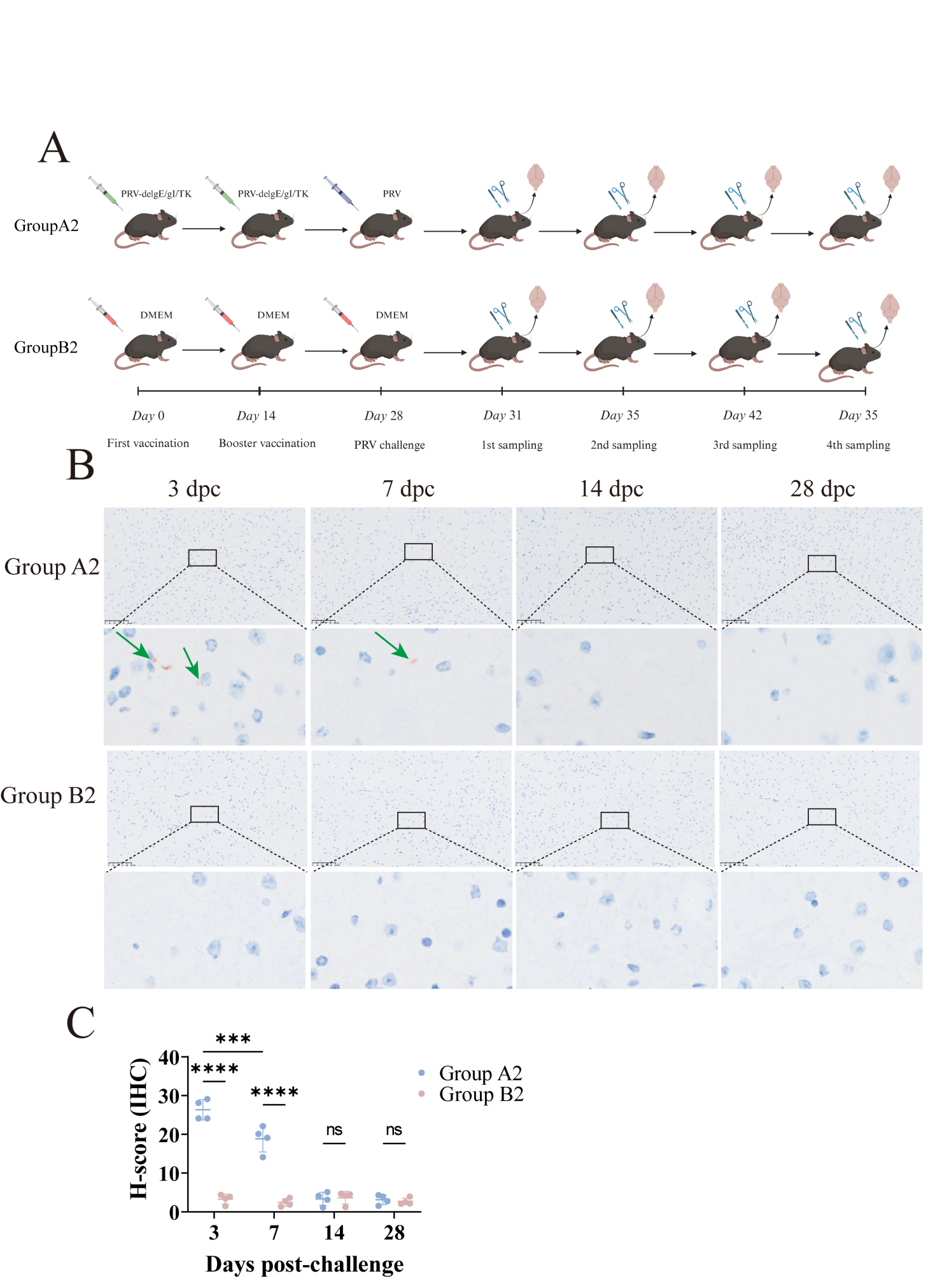


**Supplementary Fig. 1. Sampling timeline and longitudinal IHC analysis in the immunized-and-challenged and mock groups.**

(A) Schematic representation of the sampling schedule following PRV challenge. Group A2: mice immunized with PRV-delgE/gI/TK and subsequently challenged; Group B2: mock-treated controls. Brain tissues were collected at 3, 7, 14, and 28 days post-challenge for downstream analyses. (B) IHC staining of brain sections from the immunization–challenge and mock groups at 3, 7, 14, and 28 days post-challenge.Green arrows indicate regions positive for PRV gB protein.. (C) H-score quantification of IHC staining in the immunization–challenge and mock groups at 3, 7, 14, and 28 days post-challenge. *****P*<0.0001; ****P*<0.001; ***P*<0.01; **P*<0.05; ns, not significant.


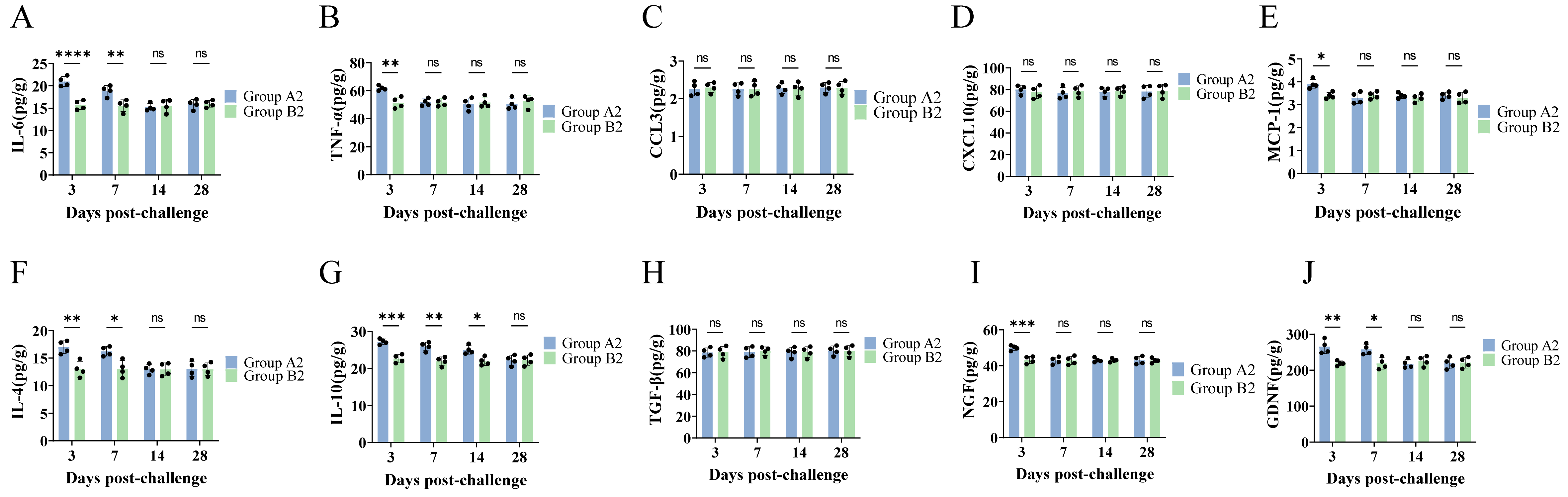


**Supplementary Fig. 2. Cytokine profiles of immunized–challenge and mock C57BL/6J mice at multiple time points (3, 7, 14, and 28 days post-challenge).**

Temporal changes in pro-inflammatory cytokines, anti-inflammatory cytokines, chemokines, and neurotrophic factors in brain tissues collected from PRV-delgE/gI/TK–immunized and mock-treated C57BL/6J mice following PRV challenge. Data are presented as mean ± SD. Statistical significance: ****p < 0.0001; ***p < 0.001; **p < 0.01; *p < 0.05; ns, not significant.
